# Lung cancer and risk of cardiovascular mortality

**DOI:** 10.3389/fcvm.2024.1491912

**Published:** 2025-01-06

**Authors:** Chengshi Wang, Zhu Wang, Jing Yang, Songbo Zhang, Purong Zhang, Ye Yang

**Affiliations:** ^1^ Department of Breast Surgery, Sichuan Clinical Research Center for Cancer, Sichuan Cancer Hospital & Institute, Sichuan Cancer Center, Affiliated Cancer Hospital of University of Electronic Science and Technology of China, Chengdu, China; ^2^ Institute for Breast Health Medicine, West China Hospital, Sichuan University, Chengdu, China; ^3^ Department of Thoracic Oncology, Sichuan Clinical Research Center for Cancer, Sichuan Cancer Hospital & Institute, Sichuan Cancer Center, Affiliated Cancer Hospital of University of Electronic Science and Technology of China, Chengdu, China

**Keywords:** lung cancer, general population, mortality, cardiovascular disease, incidence rate ratio

## Abstract

**Purpose:**

The aim of the present study was to investigate the cardiovascular mortality risk among lung cancer patients compared to the general population.

**Methods:**

Using data from the National Cancer Institute's Surveillance, Epidemiology, and End Results program, we conducted a population-based cohort study including 278,418 lung cancer patients aged over 30 years between 1 January 1990 and 31 December 2020 as well as the general population. Poisson regression was employed to calculate incidence rate ratios (IRRs) for cardiovascular mortality.

**Results:**

Patients exhibited a significantly higher IRR of cardiovascular mortality risk compared to the general population [IRR 1.74, 95% confidence interval (CI) 1.71–1.77]. The risk was most pronounced in patients aged 30–79 years (IRR 2.61, 95% CI 2.55–2.66), peaking at ages 30–34 years (IRR 48.93, 95% CI 21.98–108.92). Elevated cardiovascular mortality risks were observed across all subgroups, including diseases of the heart (IRR 1.79, 95% CI 1.75–1.82), cerebrovascular diseases (IRR 1.52, 95% CI 1.45–1.59), and other cardiovascular diseases (IRR 1.78, 95% CI 1.67–1.90). The first month after diagnosis presented the highest risk for patients aged 30–79 years (IRR 12.08, 95% CI 11.49–12.70) and ≥80 years (IRR 4.03, 95% CI 3.70–4.39). Clinical characteristics significantly modified cardiovascular mortality.

**Conclusions:**

Integrating cardiovascular disease monitoring and proactive management into lung cancer treatment protocols is essential to the improvement of overall survival and quality of life for lung cancer patients, particularly those who were young or with advanced tumor stage.

## Introduction

Lung cancer is a leading cause of cancer-related mortality worldwide, responsible for approximately 1.8 million deaths annually (1). Despite advances in early detection, surgical techniques, and systemic therapies, the prognosis for lung cancer patients remains poor, with a 5-year survival rate of approximately 20%. Lung cancer is often diagnosed at an advanced stage, complicating treatment and contributing to high mortality rates (2).

The proportion of cardiovascular disease (CVD) deaths, the leading cause of non-cancer death, accounts for approximately 5%–10% of lung cancer patients (3–5). Lung cancer and CVD share common risk factors, such as smoking, age, obesity, hypertension, diabetes, sedentary behavior, and oxidative stress (6–8). These shared risk factors contribute to an increased baseline risk of cardiovascular events and cancer mortality in lung cancer patients (9). Then, the treatments for lung cancer, including chemotherapy, radiotherapy, and targeted therapies, have cardiotoxic effects. Radiotherapy is associated with an increased risk of heart disease death in patients with lung cancer (10). Platinum and immune checkpoint inhibitors, commonly used in lung cancer treatment, are well-known for their cardiotoxicity, leading to heart failure and other cardiovascular complications (11). Newer targeted therapies, such as tyrosine kinase inhibitors, also pose risks for cardiovascular health (12). Previous studies yielded the excess risk of CVD death increased in specific lung cancer patients (e.g., higher age, male gender, squamous cell carcinoma, left-side laterality) (3, 9, 13). The cumulative burden of cancer treatment and the underlying cardiovascular risk factors necessitate a deeper understanding of cardiovascular mortality in this patient population. In addition, the inflammatory response induced by both cancer and its treatment plays a crucial role in CVD (14, 15). Moreover, the psychological stress associated with a lung cancer diagnosis can exacerbate the risk of cardiovascular mortality (16). Stress-related mechanisms, including increased sympathetic nervous system activity and elevated catecholamine levels, would precipitate cardiovascular events (17). Lung cancer patients are subjected to a multifaceted absolute risk profile that significantly elevates the risk of cardiovascular mortality. However, there is limited comprehensive study quantifying the relative risk of cardiovascular mortality in lung cancer patients compared with the general population.

The Surveillance, Epidemiology, and End Results (SEER) program provides a robust database that enables the examination of long-term trends and outcomes in individuals. By establishing a population-based cohort study including lung cancer patients and the general population, our study aims to identify age at follow-up, time since lung cancer diagnosis, and demographic and clinical factors that influence the relative risk of CVD mortality when compared with general population.

## Methods

### Patients and methods

We conducted a retrospective cohort study including patients aged over 30 years who were diagnosed with first primary lung cancer between 1 January 1990 and 31 December 2020 in the SEER database. We incorporated 53,324,676 per 100 person-years from the general population during 1990–2020 in the USA. We also selected 448,794 patients who were diagnosed with first primary lung cancer via pathological confirmation during 1990–2020. We excluded patients who were diagnosed without pathological confirmation (*N* = 48,598), whose first primary tumor was not malignant (*N* = 50), without county information (*N* = 119,217), or whose follow-up information was not calculated (*N* = 827), without race report (*N* = 486), with cancer *in situ* (*N* = 1), whose malignancy was not carcinoma (*N* = 747), and aged younger than 30 years (*N* = 450). Finally, we included 278,418 lung cancer patients.

### Certification of CVD deaths and follow-up

CVD death was defined as the primary outcome. We used the International Classification of Diseases codes (Supplementary Table S1) to confirm the death from CVD (18), which was further stratified as disease of the heart, cerebrovascular disease, or other CVD. Both patient and population follow-ups were conducted regularly using differential hospital- and population-based registries. To guarantee an adequate follow-up visit, a personal follow-up was also periodically scheduled for those who were considered lost to contact.

### Variables

Demographic information on the patients and general population was from the SEER database and the U.S. Census Bureau's Population Estimates Program, respectively, including age and calendar year at follow-up or cancer diagnosis (1990–1992, 1993–1995, 1996–1998, 1999–2001, 2002–2004, 2005–2007, 2008–2010, 2011–2015, or 2016–2020), race (white, Black, or American Indian/Alaska Native and Asian/Pacific Islander), gender (female or male), and county (counties in metropolitan areas with a population of more than 1 million, counties in metropolitan areas with a population of 250,000–1,000,000, counties in metropolitan areas with a population of less than 250,000, non-metropolitan counties not adjacent to a metropolitan area, or non-metropolitan counties adjacent to a metropolitan area). Age at follow-up, a factor generally associated with risk of CVD mortality (19), were classified into groups: 30–34 years, every 5-year interval thereafter, and older than 80 years, to adjust for the confounding impact of age. We also selected information on clinical covariates from SEER for lung cancer patients, including laterality, tumor stage, histology, grade, surgery, radiotherapy, and chemotherapy. Missing data were defined as “unknown.” The baseline characteristics of patients and the general population are shown in Table 1.

**Table 1:** Baseline characteristics of lung cancer patients and the US female population: a population-based cohort study in the USA, 1990–2020.

	Patients	Population
	Per 100 PYs (%)	Per 100 PYs (%)
Total	6,155 (100.00)	53,324,676 (100.00)
Calendar year at follow-up		
1990–1992	179 (2.90)	4,181,956 (7.84)
1993–1995	330 (5.35)	4,438,414 (8.32)
1996–1998	424 (6.89)	4,656,877 (8.73)
1999–2001	505 (8.20)	4,879,776 (9.15)
2002–2004	574 (9.32)	5,070,143 (9.51)
2005–2007	638 (10.37)	5,239,649 (9.83)
2008–2010	707 (11.48)	5,423,021 (10.17)
2011–2015	1,301 (21.13)	9,463,711 (17.75)
2016–2020	1,498 (24.35)	9,971,131 (18.70)
Age groups at follow-up (years)		
30–34	10 (0.16)	6,504,692 (12.20)
35–39	31 (0.50)	6,557,422 (12.30)
40–44	76 (1.24)	6,457,090 (12.11)
45–49	172 (2.80)	6,205,353 (11.64)
50–54	350 (5.68)	5,761,476 (10.80)
55–59	591 (9.60)	5,148,569 (9.66)
60–64	842 (13.68)	4,437,417 (8.32)
65–69	1,055 (17.14)	3,740,693 (7.01)
70–74	1,099 (17.86)	3,026,288 (5.68)
75–79	939 (15.26)	2,330,569 (4.37)
≥80	990 (16.08)	3,155,110 (5.92)
Sex		
Male	2,927 (47.55)	25,455,828 (47.74)
Female	3,228 (52.45)	27,868,848 (52.26)
Race		
White	5,151 (83.69)	44,161,940 (82.82)
Black	468 (7.61)	6,190,965 (11.61)
Other^a^	536 (8.70)	2,971,770 (5.57)
County		
Counties in metropolitan areas of larger than 1 million population	3,093 (50.26)	29,032,110 (54.44)
Counties in metropolitan areas of 250,000–1 million population	1,651 (26.82)	11,108,323 (20.83)
Counties in metropolitan areas of less than 250,000 population	480 (7.79)	4,809,260 (9.02)
Non-metropolitan counties not adjacent to a metropolitan area	445 (7.22)	2,859,368 (5.36)
Non-metropolitan counties adjacent to a metropolitan area	487 (7.91)	5,515,618 (10.34)
Laterality		
Left	2,481 (40.30)	—
Right	3,529 (57.34)	—
Bilaterality	25 (0.40)	—
Unknown	120 (1.95)	—
Time since diagnosis		
0 to <1 month	217 (3.53)	—
1 to <6 month	810 (13.16)	—
6 to <12 month	711 (11.55)	—
1 to <2 years	931 (15.12)	—
2 to <5 years	1,530 (24.86)	—
5 to <10 years	1,182 (19.21)	—
≥10 years	774 (12.57)	—
Histology		
NSCLC		
Adenocarcinoma	2,993 (48.63)	—
Squamous cell carcinoma	1,306 (21.22)	—
Others	780 (12.67)	—
Unclassified	327 (5.31)	—
SCLC	509 (8.28)	—
Carcinoma unclassified	239 (3.89)	—
Tumor grade		
Well differentiated	623 (10.12)	—
Moderately differentiated	1,439 (23.38)	—
Poorly differentiated	1,626 (26.41)	—
Undifferentiated	357 (5.80)	—
Unknown	2,110 (34.28)	—
Tumor stage		
Local	2,530 (41.10)	—
Regional	2,059 (33.46)	—
Distant	1,369 (22.25)	—
Unknown	196 (3.19)	—
Surgery		
No	2,493 (40.51)	—
Yes	3,642 (59.17)	—
Unknown	20 (0.33)	—
Radiation		
No/unknown	2,071 (33.65)	—
Yes	4,084 (66.35)	—
Chemotherapy		
No/unknown	3,889 (63.18)	—
Yes	2,266 (36.82)	—

CI, confidence interval; PYs, person-years; NSCLC, non-small cell lung cancer; SCLC, small cell lung cancer.

^a^ American Indian/Alaska Native and Asian/Pacific Islander.

### Statistical analysis

Using Poisson regression, we calculated the incidence rate ratios (IRRs) and 95% confidence intervals (CIs) of CVD deaths among patients relative to the general population controlling for age at follow-up, race, gender, county, and calendar year at follow-up. We described the cumulative mortality rate for lung cancer patients aged 80 years at follow-up. We estimated that IRRs of death due to heart diseases, cerebrovascular diseases, and other CVDs in subgroups. We also assessed IRRs by follow-up time since cancer diagnosis. We conducted subsequent subgroup analyses in patients aged 30–79 years at follow-up, on account of the obviously increased risk in this age group.

STATA (version 16.0; Stata Corporation) was used to calculate statistical analyses. *p* < 0.05 indicates statistical significance.

## Results

### Survival characteristics

Survival data of lung cancer patients and the general population were obtained from the SEER database and the U.S. Census Bureau's Population Estimates Program between 1990 and 2020, respectively. A total of 278,418 lung cancer patients were diagnosed during 1990–2020, with 12,584 CVD deaths (mortality rate: 2.04 per 100 person-years) and a median follow-up of 9 months (interquartile range 3–27 months). In total, 26,948,910 CVD deaths (mortality rate: 0.51 per 100 person-years) were identified in the general population.

### Age at follow-up

**Figure 1:**
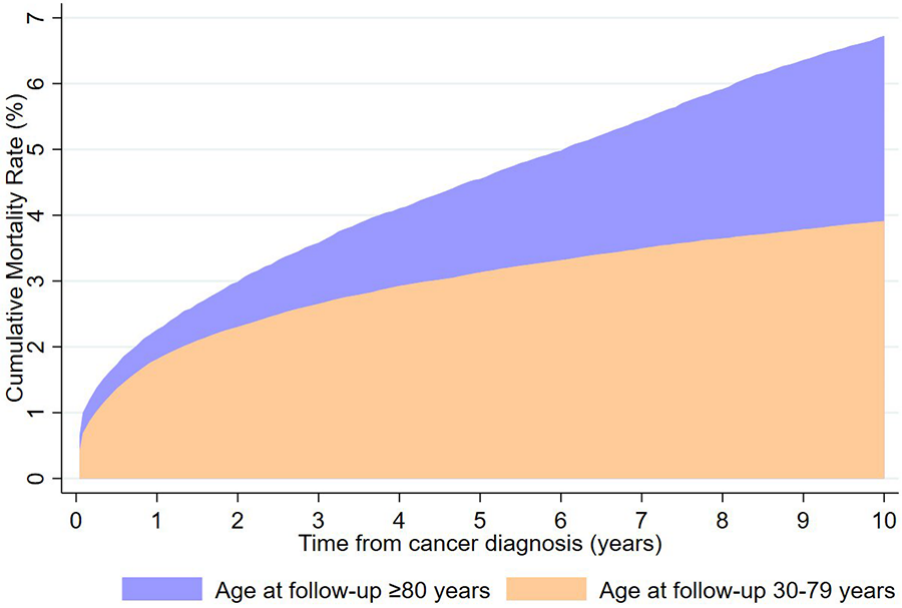
Cumulative mortality rates of cardiovascular death by age at follow-up from lung cancer diagnosis to 10 years.

The cumulative mortality rate of CVD among lung cancer survivors aged over 80 years at follow-up was significantly higher than that of those aged 30–79 years a decade after diagnosis (6.73% vs. 3.91%; Figure 1).

Overall, lung cancer patients had a higher IRR of CVD mortality compared with the general population (IRR 1.74, 95% CI 1.71–1.77) (Table 2). Furthermore, patients aged 30–79 years at follow-up were correlated with a more significantly increased risk of CVD mortality (IRR 2.61, 95% CI 2.55–2.66) (Table 2) and this correlation was most pronounced among those aged 30–34 years (IRR 48.93, 95% CI 21.98–108.92) (Table 2).

**Table 2:** Adjusted IRRs for cardiovascular mortality among lung cancer patients compared to the general population, stratified by age group: a population-based cohort study in the USA, 1990–2020.

	Population *N* (MR)	Patients *N* (MR)	IRR (95% CI)^a^
Overall	26,948,910 (0.51)	12,584 (2.04)	1.74 (1.71–1.77)
Age groups at follow-up (years)			
30–79	12,696,829 (0.25)	8,353 (1.62)	2.61 (2.55–2.66)
30–34	85,130 (0.01)	6 (0.60)	48.93 (21.98–108.92)
35–39	162,402 (0.02)	10 (0.33)	14.07 (7.57–26.16)
40–44	304,751 (0.05)	29 (0.38)	8.53 (5.92–12.27)
45–49	520,887 (0.08)	106 (0.62)	7.64 (6.32–9.25)
50–54	803,370 (0.14)	254 (0.73)	5.35 (4.73–6.05)
55–59	1,131,618 (0.22)	574 (0.97)	4.53 (4.17–4.91)
60–64	1,540,194 (0.35)	1,039 (1.23)	3.65 (3.43–3.88)
65–69	2,010,721 (0.54)	1,681 (1.59)	3.10 (2.95–3.25)
70–74	2,661,866 (0.88)	2,156 (1.96)	2.41 (2.31–2.51)
75–79	3,475,890 (1.49)	2,498 (2.66)	1.96 (1.89–2.04)
≥80	14,252,081 (4.52)	4,231 (4.27)	1.05 (1.02–1.08)
P for interaction^b^			<0.001

CI, confidence interval; IRR, incidence rate ratio; MR, mortality rate per 100 person-years; *N*, number of deaths.

Data were obtained from the SEER database between 1990 and 2020. Poisson regression was used for risk estimation.

^a^ IRRs were adjusted for age at follow-up (30–34 years, every 5 years thereafter, or ≥80 years), sex (female or male), race (white, Black, or other), county (counties in metropolitan areas with a population larger than 1 million, counties in metropolitan areas with a population of 250,000–1,000,000, counties in metropolitan areas with a population of less than 250,000, non-metropolitan counties not adjacent to a metropolitan area, or non-metropolitan counties adjacent to a metropolitan area), and calendar year at follow-up (1990–1992, 1993–1995, 1996–1998, 1999–2001, 2002–2004, 2005–2007, 2008–2010, 2011–2015, or 2016–2020).

^b^ We added an interaction between lung cancer and age at follow-up (30–34 years, every 5 years thereafter, or ≥80 years) and reported the significance level of the term as *p* for interaction.

In the subgroup analysis, lung cancer patients aged 30–79 years at follow-up showed a greater risk association for deaths from heart of diseases, cerebrovascular diseases, and other CVDs compared to the general population (Table 3).

**Table 3:** IRRs of cardiovascular mortality in lung cancer patients compared to the general population by subgroup: a population-based study in the USA, 1990–2020.

	Population *N* (MR)	Patients *N* (MR)	IRR (95% CI)^a^
Diseases of heart			
Overall	20,669,892 (0.39)	9,834 (1.60)	1.79 (1.75–1.82)
Age at follow-up (years)			
30–79	10,027,554 (0.20)	6,555 (1.27)	2.62 (2.56–2.69)
≥80	10,642,337 (3.37)	3,279 (3.31)	1.09 (1.05–1.13)
Cerebrovascular diseases			
Overall	4,523,101 (0.09)	1,882 (0.31)	1.52 (1.45–1.59)
Age at follow-up (years)			
30–79	1,869,634 (0.04)	1,207 (0.23)	2.44 (2.31–2.58)
≥80	2,653,467 (0.84)	675 (0.68)	0.91 (0.84–0.98)
Other cardiovascular diseases			
Overall	1,743,190 (0.03)	868 (0.14)	1.78 (1.67–1.90)
Age at follow-up (years)			
30–79	788,329 (0.02)	591 (0.11)	2.80 (2.59–3.04)
≥80	954,861 (0.30)	277 (0.28)	1.00 (0.89–1.12)

CI, confidence interval; IRR, incidence rate ratio; MR, mortality rate per 100 person-years; *N*, number of deaths.

Data were obtained from the SEER database between 1990 and 2020. Poisson regression was used for risk estimation.

^a^ IRRs were adjusted for age at follow-up (30–34 years, every 5 years thereafter, or ≥80 years), sex (female or male), race (white, Black, or other), county (counties in metropolitan areas with a population larger than 1 million, counties in metropolitan areas with a population of 250,000–1,000,000, counties in metropolitan areas with a population of less than 250,000, non-metropolitan counties not adjacent to a metropolitan area, or non-metropolitan counties adjacent to a metropolitan area), and calendar year at follow-up (1990–1992, 1993–1995, 1996–1998, 1999–2001, 2002–2004, 2005–2007, 2008–2010, 2011–2015, or 2016–2020).

### Demographic and clinical characteristics

We performed subsequent analyses by restricting patients aged 30–79 years, as increased risk was significant in this age group. A higher magnitude of associations was found among lung cancer patients who were female (IRR 2.82, 95% CI 2.72–2.92), who lived in counties in metropolitan areas with a population of 250,000–1,000,000 people (IRR 2.94, 95% CI 2.83–3.06), earlier calendar year at follow-up (1990–1992, IRR 3.75, 95% CI 3.46–4.07), who had small cell lung cancer (SCLC) (IRR 3.53, 95% CI 3.32–3.77), who had bilateral carcinoma (IRR 3.82, 95% CI 2.91–5.02), metastasis (IRR 4.03, 95% CI 3.88–4.19), who had an undifferentiated tumor grade (IRRs 3.12, 95% CI 2.89–3.37), or who did not receive chemotherapy, radiation, or surgery (IRR 2.66–3.90) (Tables 4, 5). In addition, we also found that lung cancer patients who received chemotherapy (IRR 2.50, 95% CI 2.41–2.60) or radiotherapy (IRR 2.44, 95% CI 2.37–2.51) exhibited significantly higher risks of cardiovascular mortality compared to the general population (Table 5).

**Table 4:** IRRs of cardiovascular mortality in lung cancer patients aged 30–79 years at follow-up compared to the general population, stratified by demographic characteristics: a population-based study in the USA, 1990–2020.

	Population *N* (MR)	Patients *N* (MR)	IRR (95% CI)^a^
Gender			
Male	7,753,770 (0.32)	5,177 (2.06)	2.49 (2.42–2.56)
Female	4,943,059 (0.19)	3,176 (1.20)	2.82 (2.72–2.92)
*p* for interaction			<0.001
Race			
White	10,337,283 (0.25)	7,017 (1.63)	2.62 (2.56–2.68)
Black	2,049,959 (0.34)	771 (1.80)	2.08 (1.94–2.23)
Other^b^	309,587 (0.11)	565 (1.29)	3.60 (3.31–3.91)
*p* for interaction			<0.001
County			
Counties in metropolitan areas of larger than 1 million population	6,254,924 (0.23)	3,882 (1.49)	2.51 (2.44–2.59)
Counties in metropolitan areas of 250,000–1 million population	2,643,129 (0.25)	2,408 (1.76)	2.94 (2.83–3.06)
Counties in metropolitan areas of less than 250,000 population	1,253,674 (0.28)	604 (1.50)	2.31 (2.13–2.50)
Non-metropolitan counties not adjacent to a metropolitan area	859,567 (0.32)	686 (1.85)	2.47 (2.30–2.67)
Non-metropolitan counties adjacent to a metropolitan area	1,685,535 (0.33)	773 (1.88)	2.54 (2.36–2.72)
*p* for interaction			<0.001
Calendar year at follow-up			
1990–1992	1,434,103 (0.36)	580 (3.50)	3.75 (3.46–4.07)
1993–1995	1,415,328 (0.34)	791 (2.62)	2.90 (2.70–3.11)
1996–1998	1,364,058 (0.31)	907 (2.38)	2.81 (2.63–3.00)
1999–2001	1,290,814 (0.28)	889 (2.00)	2.59 (2.42–2.76)
2002–2004	1,201,677 (0.25)	930 (1.89)	2.77 (2.60–2.95)
2005–2007	1,098,103 (0.22)	831 (1.55)	2.61 (2.44–2.79)
2008–2010	1,044,129 (0.21)	791 (1.37)	2.59 (2.41–2.77)
2011–2015	1,792,370 (0.20)	1,281 (1.22)	2.46 (2.33–2.59)
2016–2020	2,056,247 (0.22)	1,353 (1.12)	2.15 (2.04–2.26)
*p* for interaction			<0.001

CI, confidence interval; N, number of death; MR, mortality rate per 100 person-years.

Data were obtained from the SEER database between 1990 and 2020. Poisson regression was used for risk estimation.

^a^ IRRs were adjusted for age at follow-up (30–34 years, every 5 years thereafter, or ≥80 years), sex (female or male), race (white, Black, or other), county (counties in metropolitan areas with a population larger than 1 million, counties in metropolitan areas with a population of 250,000–1,000,000, counties in metropolitan areas with a population of less than 250,000, non-metropolitan counties not adjacent to a metropolitan area, or non-metropolitan counties adjacent to a metropolitan area), and calendar year at follow-up (1990–1992, 1993–1995, 1996–1998, 1999–2001, 2002–2004, 2005–2007, 2008–2010, 2011–2015, or 2016–2020). The interaction term of IRRs across demographic characteristics was added and the *p*-value for significance was reported.

^b^ American Indian/Alaska Native and Asian/Pacific Islander.

**Table 5:** IRRs of cardiovascular mortality in lung cancer patients aged 30–79 years at follow-up compared to the general population, by clinical characteristics: a population-based study in the US, 1990–2020.

	Patients *N* (MR)	IRR (95% CI)^a^
Laterality		
Left	5315 (2.14)	2.63 (2.55–2.72)
Right	6826 (1.93)	2.51 (2.44–2.59)
Bilaterality	71 (2.88)	3.82 (2.91–5.02)
*p* for difference		<0.001
Histology		
NSCLC	10,577 (1.96)	2.45 (2.39–2.51)
Adenocarcinoma (NSCLC)	5,123 (1.71)	2.23 (2.16–2.31)
Squamous cell carcinoma (NSCLC)	3,506 (2.68)	2.73 (2.63–2.85)
Others (NSCLC)	1,117 (1.43)	2.00 (1.86–2.16)
Unclassified (NSCLC)	831 (2.54)	3.89 (3.59–4.22)
SCLC	1,163 (2.28)	3.53 (3.32–3.77)
Carcinoma unclassified	844 (3.52)	3.84 (3.54–4.17)
*p* for difference		<0.001
Tumor stage		
Local	4,504 (1.78)	1.92 (1.85–2.00)
Regional	3,938 (1.91)	2.48 (2.39–2.57)
Distant	3,469 (2.53)	4.03 (3.88–4.19)
Unstaged	673 (3.43)	3.42 (3.11–3.77)
*p* for difference		<0.001
Tumor grade		
Well differentiated	866 (1.39)	1.57 (1.43–1.73)
Moderately differentiated	2,457 (1.71)	2.01 (1.91–2.11)
Poorly differentiated	3,451 (2.12)	2.57 (2.47–2.68)
Undifferentiated	849 (2.38)	3.12 (2.89–3.37)
*p* for difference		<0.001
Chemotherapy		
No/unknown	9,281 (2.39)	2.66 (2.59–2.73)
Yes	3,303 (1.46)	2.50 (2.41–2.60)
*p* for difference		<0.001
Radiotherapy		
No/unknown	4,357 (2.10)	2.93 (2.83–3.03)
Yes	8,227 (2.01)	2.44 (2.37–2.51)
*p* for difference		<0.001
Surgery		
No/unknown	7,274 (2.89)	3.90 (3.80–4.01)
Yes	5,310 (1.46)	1.71 (1.66–1.77)
*p* for difference		<0.001

CI, confidence interval; PYs, person-years; *N*, number of death; MR, mortality rate per 100 person-years; NSCLC, non-small cell lung cancer; SCLC, small cell lung cancer.

The population was regarded as reference [*N* (MR):26,948,910 (0.51)] in every estimate. Data were obtained from the SEER database between 1990 and 2020. Poisson regression was used for risk estimation.

^a^ IRRs were adjusted for age at follow-up (30–34 years, every 5 years thereafter, or ≥80 years), sex (female or male), race (white, Black, or other), county (counties in metropolitan areas with a population larger than 1 million, counties in metropolitan areas with a population of 250,000–1,000,000, counties in metropolitan areas of less than 250,000, non-metropolitan counties not adjacent to a metropolitan area, or non-metropolitan counties adjacent to a metropolitan area), and calendar year at follow-up (1990–1992, 1993–1995, 1996–1998, 1999–2001, 2002–2004, 2005–2007, 2008–2010, 2011–2015, or 2016–2020). These IRRs were estimated by comparing clinical characteristics among lung cancer patients with the general population. The difference of IRRs across characteristics was performed using the Wald test and the *p*-value for significance was reported.

### Follow-up time since lung cancer diagnosis

**Figure 2:**
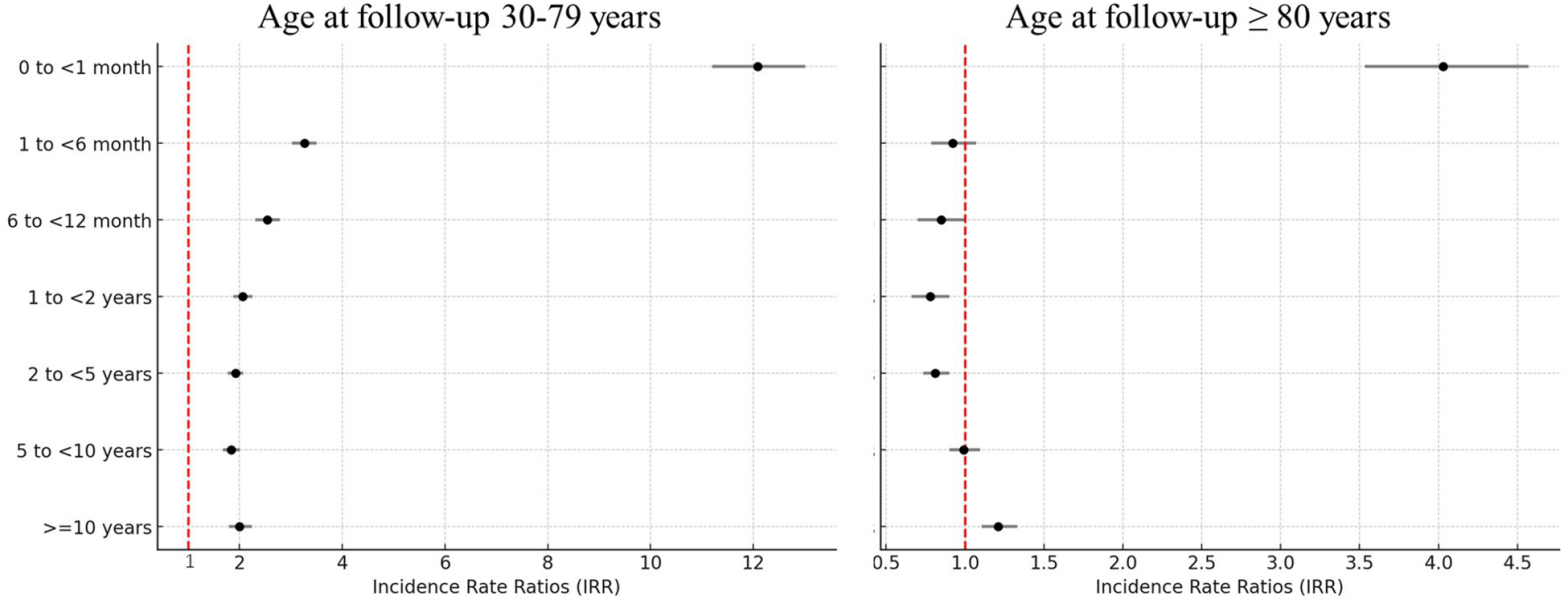
IRRs of cardiovascular mortality by time since cancer diagnosis, comparing lung cancer patients with the general population: a population-based study in the USA, 1990–2020. IRRs were adjusted for age at follow-up (30–34 years, every 5 years thereafter, or ≥80 years), sex (female or male), race (white, Black, or other), county (counties in metropolitan areas with populations larger than 1 million, counties in metropolitan areas with a population of 250,000–1,000,000 population, counties in metropolitan areas with a population of less than 250,000, non-metropolitan counties not adjacent to a metropolitan area, or non-metropolitan counties adjacent to a metropolitan area), and calendar year at follow-up (1990–1992, 1993–1995, 1996–1998, 1999–2001, 2002–2004, 2005–2007, 2008–2010, 2011–2015, or 2016–2020).

The risks of CVD mortality were greatest among lung cancer patients aged 30–79 years and ≥80 years at follow-up, respectively, during the first month after lung cancer diagnosis (IRR 12.08, 95% CI 11.49–12.70; and IRR 4.03, 95% CI 3.7–4.39) (Figure 2 and statistical values in Supplementary Table S2) compared with the general population.

## Discussion

The population-based cohort study demonstrates that lung cancer patients face a significantly higher risk of cardiovascular mortality compared to the general population. The risk peaks during the first month after lung cancer diagnosis and is particularly pronounced in younger patients. A study reported that patients with lung cancer had a standardized mortality ratio (SMR) of 7–14 for fatal heart disease within the first year after diagnosis, with this elevated trend persisting throughout all follow-up periods (20). Our findings indicate that early diagnosis of lung cancer is strongly associated with a significantly increased risk of CVD mortality, particularly within the first month after diagnosis. During this critical period, patients in the younger (aged 30–79 years; IRR 12.08, 95% CI 11.49–12.70) and older (aged ≥80 years; IRR 4.03, 95% CI 3.70–4.39) groups exhibit the greatest increased risk of CVD mortality compared with the general population. Several factors likely contribute to this early and heightened cardiovascular risk. First, being diagnosed with lung cancer, a life-threatening illness, can induce significant psychological stress, which is linked to adverse cardiovascular outcomes (17). Stress-related mechanisms include increased sympathetic nervous system activity and higher levels of circulating catecholamines, which can precipitate cardiovascular events such as myocardial infarction and stroke (21, 22). Second, the initiation of cancer treatments may exacerbate pre-existing cardiovascular conditions. Surgery induces significant perioperative stress, increasing the risk of thromboembolism and arrhythmias (23). Similarly, chemotherapy (tyrosine kinase inhibitors) and immunotherapy are associated with acute myocardial injury, while radiation therapy can induce vascular inflammation and fibrosis (12, 24–26). Third, systemic inflammation induced by both cancer progression and its treatment plays a critical role. Pro-inflammatory cytokines, such as IL-6 and TNF-α, are significantly elevated in lung cancer patients and contribute to endothelial dysfunction, plaque instability, and increased coagulation, all of which heighten cardiovascular risk (27, 28). These findings underscore the importance of close cardiovascular monitoring after a diagnosis of lung cancer, particularly for younger patients. Tailored cardio-oncology care strategies, including baseline cardiovascular risk assessment and early intervention, could significantly mitigate this heightened risk and improve patient outcomes. In addition, temporal analysis reveals a decline in cardiovascular mortality risk over the study period (1990–2020), particularly from 2010 onward, coinciding with the introduction of immunotherapy and advanced chemotherapy protocols. These findings highlight the impact of evolving treatment strategies on cardiovascular outcomes (29).

Radiation therapy may elevate the risk of CVD mortality by triggering acute inflammatory cascades, which can lead to myocardial fibrosis and subsequent damage to cardiac muscle and surrounding vasculature (25). Chemotherapy, particularly tyrosine kinase inhibitors, also leads to a higher risk of CVD mortality due to injury of the circulation system (11). Our findings of an increased risk of CVD mortality in lung cancer patients who received radiation/chemotherapy further validate this association. Although female patients with lung cancer have a lower absolute risk of CVD-specific death relative to male patients (3), they exhibit a relatively higher risk when compared to the general female population. This may be due to a higher frequency of cardiotoxicity observed in women undergoing cancer treatment (25, 30). Cardiotoxicity from lung cancer treatment extends beyond left ventricular dysfunction, which is often a late-stage manifestation of cardiac damage. Emerging evidence highlights the utility of more sensitive metrics for early detection. Techniques such as myocardial strain analysis via echocardiography and biomarkers like cardiac troponins and Brain Natriuretic Peptide (BNP) offer valuable insights into subclinical cardiac injury (31). These measures can detect myocardial damage long before symptoms appear or left ventricular ejection fraction declines, thus allowing for the timely alleviation of the cumulative cardiovascular burden of lung cancer treatment and reduction of long-term complications. In addition to treatment-related cardiotoxicity, gender differences in CVD mortality among lung cancer patients are also influenced by biological and socio-behavioral factors. Biologically, hormonal factors, such as the cardioprotective effects of estrogen, might partially explain the lower absolute CVD mortality risk in women compared to men (32). In male patients, coronary artery disease primarily affects the epicardial coronary arteries, whereas in female patients, it is the microvascular circulation that is most significantly impacted, which may increase women's susceptibility to cardiotoxic effects (33). Socio-behavioral factors also play a role. Tobacco use, obesity, type 2 diabetes mellitus, depression, and psychosocial stress have a more powerful impact on CVD on female patients than they do on male patients (33). Addressing these disparities requires a gender-sensitive approach to cardio-oncology care, including proactive cardiovascular monitoring and tailored interventions for women undergoing cancer treatment. Patients with poorly differentiated tumors or bilateral lung cancer face a higher risk of CVD mortality due to complex treatment methods (24), heavy tumor burden, systemic inflammation, and metabolic disorders (34). Our analysis suggests advanced-stage lung cancer correlates with higher CVD mortality, highlighting the importance of cardiovascular management, particularly for younger patients and those who have not undergone treatment. Moreover, our findings demonstrate that patients with SCLC exhibit a significantly higher cardiovascular mortality risk (IRR 3.53, 95% CI: 3.32–3.77) than both the general population and patients with non-small cell lung cancer (NSCLC). The aggressive nature of SCLC leads to intense treatment regimens, including high-dose chemotherapy and thoracic radiotherapy, both of which are known to have cardiotoxic effects (35). Rapid tumor progression and higher baseline systemic inflammation in SCLC patients may further exacerbate cardiovascular mortality risks (36).

Aging is a significant factor in both CVD and lung cancer risk (5, 9). Our findings indicate that CVD mortality rates increase with age in both lung cancer patients and the general population, and that absolute CVD mortality rates increase with age in both lung cancer patients and the general population. Interestingly, in comparison with the general population, our study shows a dramatic increase in relative CVD mortality risk among patients aged 30–34 years, while those aged 80 years and older experience a milder increase. This aligns with recent findings suggesting that younger cancer survivors face a disproportionately higher risk of heart disease-related death compared to their age-matched counterparts in the general population (5).

Clinically, this study provides actionable insights for improving patient care. It underscores the necessity of integrating cardiovascular risk assessment and management into lung cancer treatment protocols, particularly during the first month after diagnosis and for younger or high-risk subgroups. By identifying specific demographic and clinical factors associated with increased CVD mortality, this research offers a roadmap for tailoring cardio-oncology interventions to enhance patient outcomes. In addition, this study serves as a call to action for future research. It highlights the need to explore the mechanisms underlying age- and temporal-specific differences in cardiotoxicity, investigate the long-term impact of cancer therapies on cardiovascular health, and develop preventive strategies that balance cancer treatment efficacy with cardiovascular safety.

## Limitations

Some limitations should be noted in this study. First, the available information on the general population is not cancer-free, which may contribute to underestimating the actual risk of CVD mortality. A second limitation is the lack of data on comorbid conditions, such as hypertension and diabetes. These conditions are known to exacerbate cardiovascular risks, particularly in lung cancer patients undergoing aggressive treatment. Still and all, the significantly elevated risk of CVD mortality within 1 month after a cancer diagnosis alleviates this concern. Finally, although the distribution characteristics of lung cancer patients and the general population in age at follow-up are unbalanced, we still investigated the analyzable association by age between the two groups.

## Conclusions

Our results show lung cancer patients are at an increased risk of CVD mortality compared with the general population. These findings underscore the importance of monitoring CVD in lung cancer patients, especially those who are younger or in advanced stages. The greatest risk of CVD mortality occurs during the first month after lung cancer diagnosis, suggesting the need for tailored CVD care strategies.

## Data Availability

The datasets presented in this study can be found in online repositories. The names of the repository/repositories and accession number(s) can be found below: Surveillance, Epidemiology, and End Results Program (SEER) database.
