# Supplementary material for: Lung cancer and risk of cardiovascular mortality
Source: Front Cardiovasc Med. 2025 Jan 6;11:1491912. doi: 10.3389/fcvm.2024.1491912 (PMC11743495; doi:10.3389/fcvm.2024.1491912)
Supplement: Supplementary file 1 [file Table1.doc]

Table S1. International Classification of Diseases (ICD) codes identifying deaths from cardiovascular disease including disease of the heart, cerebrovascular disease, or other cardiovascular disease.

Death of cardiovascular disease	ICD-8 (1968-1978)	ICD-9 (1979-1998)	ICD-10 (1999-present)	
Cardiovascular disease	390-448	390-448	I00–I78	
Subgroup				
  Disease of the heart	390-398, 402, 404, 410-429	390-398, 402, 404, 410-429	I00-I09, I11, I13, I20-I51	
  Cerebrovascular disease	430-438	430-438	I60-I69	
  Other cardiovascular disease	The remaining codes	


 
