# Supplementary material for: Lung cancer and risk of cardiovascular mortality
Source: Front Cardiovasc Med. 2025 Jan 6;11:1491912. doi: 10.3389/fcvm.2024.1491912 (PMC11743495; doi:10.3389/fcvm.2024.1491912)
Supplement: Supplementary file 2 [file Table2.docx]

**Table S2. Incidence rate ratios (IRRs) of cardiovascular mortality in lung cancer patients by time since cancer diagnosis, compared with the general population.**

|  | **Patients**  **N (MR)** | **IRR (95% CI)**^a^ |
| --- | --- | --- |
| **Age at follow up 30 to 79 years** | | |
| **By time since diagnosis** |  |  |
| 0 to <1 month | 1,539 (8.20) | 12.08 (11.49-12.70) |
| 1 to <6 month | 1,505 (2.11) | 3.26 (3.10-3.42) |
| 6 to <12 month | 980 (1.55) | 2.54 (2.38-2.70) |
| 1 to <2 years | 1,030 (1.26) | 2.06 (1.94-2.19) |
| 2 to <5 years | 1,531 (1.17) | 1.92 (1.82-2.02) |
| 5 to <10 years | 1,078 (1.14) | 1.84 (1.73-1.95) |
| >10 years | 690 (1.23) | 2.00 (1.86-2.16) |
| **Age at follow-up ≥80 years** | | |
| **By time since diagnosis** |  |  |
| 0 to <1 month | 532 (17.86) | 4.03 (3.70-4.39) |
| 1 to <6 month | 384 (4.03) | 0.92 (0.83-1.02) |
| 6 to <12 month | 278 (3.60) | 0.85 (0.75-0.95) |
| 1 to <2 years | 372 (3.26) | 0.78 (0.70-0.86) |
| 2 to <5 years | 755 (3.33) | 0.81 (0.76-0.87) |
| 5 to <10 years | 929 (3.98) | 0.99 (0.93-1.06) |
| >10 years | 981 (4.60) | 1.21 (1.14-1.29) |

Abbreviations: CI, confidence interval; IRR, incidence-rate ratio; MR, mortality rate per 100 person-years; N, number of deaths.

^a^ IRRs were adjusted for age at follow up (30-34, every 5 years afterwards, or ≥80 years), sex(female or male), race (white, black, or other), county(Counties in metropolitan areas of larger than 1 million population, Counties in metropolitan areas of 250,000 to 1 million population, Counties in metropolitan areas of less than 250 thousand population, Nonmetropolitan counties not adjacent to a metropolitan area or Nonmetropolitan counties adjacent to a metropolitan area), and calendar year at follow-up (1990-1992, 1993-1995, 1996-1998, 1999-2001, 2002-2004, 2005-2007, 2008-2010, 2011-2015 or 2016-2020).
